# A Two-Stage Model for Predicting Mild Cognitive Impairment to Alzheimer’s Disease Conversion

**DOI:** 10.3389/fnagi.2022.826622

**Published:** 2022-03-21

**Authors:** Peixin Lu, Lianting Hu, Ning Zhang, Huiying Liang, Tao Tian, Long Lu

**Affiliations:** ^1^School of Information Management, Wuhan University, Wuhan, China; ^2^Medical Big Data Center, Guangdong Provincial People’s Hospital, Guangzhou, China; ^3^Guangdong Cardiovascular Institute, Guangdong Provincial People’s Hospital, Guangzhou, China; ^4^School of Business, Qingdao University, Qingdao, China; ^5^The First Division of Psychiatry, Jingmen No. 2 People’s Hospital, Jingmen, China

**Keywords:** mild cognitive impairment, Alzheiemer’s disease, contrastive learning, transfer leaning, MRI, deep learning

## Abstract

Early detection of Alzheimer’s disease (AD), such as predicting development from mild cognitive impairment (MCI) to AD, is critical for slowing disease progression and increasing quality of life. Although deep learning is a promising technique for structural MRI-based diagnosis, the paucity of training samples limits its power, especially for three-dimensional (3D) models. To this end, we propose a two-stage model combining both transfer learning and contrastive learning that can achieve high accuracy of MRI-based early AD diagnosis even when the sample numbers are restricted. Specifically, a 3D CNN model was pretrained using publicly available medical image data to learn common medical features, and contrastive learning was further utilized to learn more specific features of MCI images. The two-stage model outperformed each benchmark method. Compared with the previous studies, we show that our model achieves superior performance in progressive MCI patients with an accuracy of 0.82 and AUC of 0.84. We further enhance the interpretability of the model by using 3D Grad-CAM, which highlights brain regions with high-predictive weights. Brain regions, including the hippocampus, temporal, and precuneus, are associated with the classification of MCI, which is supported by the various types of literature. Our model provides a novel model to avoid overfitting because of a lack of medical data and enable the early detection of AD.

## Introduction

Alzheimer’s disease (AD), a severe neurodegenerative disease, is the most common type of dementia (Heun et al., 1997; Association, 2019). Nowadays, at least 50 million people worldwide suffer from AD or other types of dementia, and it is expected that this number will reach 131 million in 2050 (Livingston et al., 2017). This further increases the burden of the medical care system in aging societies. Mild cognitive impairment (MCI) is a stage between normal and AD, with 10–12% of people developing AD each year (Petersen, 2000). Based on the progression toward AD, it can be classified into two categories: progressive MCI (pMCI) and stable MCI (sMCI). Although there is no effective treatment for AD at present, its progression can be slowed by medication, memory training, exercise, and diet, which necessitates the early detection of potential patients (Roberson and Mucke, 2006). Neuroimaging techniques, which can detect disease-related neuropathological changes, are valuable tools for assessing and diagnosing AD (Johnson et al., 2012). MRI is one of the most widely studied neuroimaging techniques because it is non-invasive, generally available, affordable, and capable of distinguishing between different soft tissues (Klöppel et al., 2008).

With the rapid development, deep learning has achieved remarkable progress in a variety of fields, especially in computer vision and medical imaging, where it outperforms traditional machine learning methods (Shen et al., 2017; Bernal et al., 2019; Abrol et al., 2021). Deep learning approaches perform feature selection during model training and loss function optimization without the need for domain experts’ prior knowledge. As a result, individuals with no medical expertise can use them for research and applications, especially in the field of medical image analysis (Shen et al., 2017). Notably, Convolutional Neural Network (CNN) has achieved outstanding performance in the classification tasks of AD and normal control (NC) (Liu et al., 2019) and pMCI/sMCI (Choi and Jin, 2018; Spasov et al., 2019). In general, deep neural networks require large samples for model fitting, especially 3D deep neural network models with more parameters. However, as compared with existing million-sample natural image datasets, neuroimaging datasets have a relatively small sample size (Russakovsky et al., 2015), which can possibly be explained by the following factors. At first, collecting large training sets and labeling image data are costly and time consuming (Shen et al., 2017; Irvin et al., 2019). Furthermore, technical and privacy issues also constraints obstruct medical data collection (Irvin et al., 2019). Therefore, preventing model overfitting due to the scarcity of medical samples has become one of the hottest topics in deep learning of neuroimaging.

Transfer learning is a popular method for dealing with a small number of samples. It utilizes a pretrained model with supervised learning on a large labeled dataset (source domain, e.g., ImageNet) and then fine tunes it on the task of interest (target domain). Studies have shown that knowledge transferred from large-scale annotated natural images (ImageNet) to medical images can significantly improve the effectiveness of assisted diagnosis (Tajbakhsh et al., 2016; Raghu et al., 2019). However, standard medical images, such as MRI, CT, and positron emission tomography (PET), are in three dimensional (3D), preventing ImageNet-based pretrained models from being directly transferred to MRI. Converting 3D data into two-dimensional (2D) slices is a typical method, however, this ignores the rich 3D spatial anatomical information and inevitably affects the performance. To address this issue, several studies (Yang et al., 2017; Zeng and Zheng, 2018) have used pretrained 3D models based on natural video datasets (Tran et al., 2014; Carreira and Zisserman, 2017) to transfer to medical imaging tasks, but have not yet achieved the expected results because of the vast difference between these two domains.

Recently, contrastive learning, a self-supervised learning method, has recently been demonstrated to perform superiorly in various vision tasks (Wu et al., 2018; Zhuang C. et al., 2019; Chen X. et al., 2020). Momentum Contrast (MoCo) (He et al., 2020) is a state-of-the-art method in contrastive learning, which minimizes positive pairs variability while maximizing negative pairs variability. Based on existing research concerns, we proposed a two-stage model based on MoCo (He et al., 2020) to classify sMCI and pMCI. The main contributions of our study are as follows.

1) Systematic evaluation of 3D ResNet models with different structures and selection of the best model for sMCI and pMCI classification. Provides a reference for related studies.
2) A two-stage model is proposed to solve the problem of domain transfer between the source and target domains, which solves the problem of overfitting caused by small samples in sMCI and pMCI classification and improves the classification performance in AD diagnosis. To the best of our knowledge, we first introduce the MoCo in pMCI and sMCI classification.
3) Three-dimensional Gradient-weighted Class Activation Mapping (Grad-CAM), which is widely used for model interpretability, was introduced to get the heatmap that highlights the brain regions our model focuses on and increases the interpretability of the model.

## Materials and Methods

**FIGURE 1:**
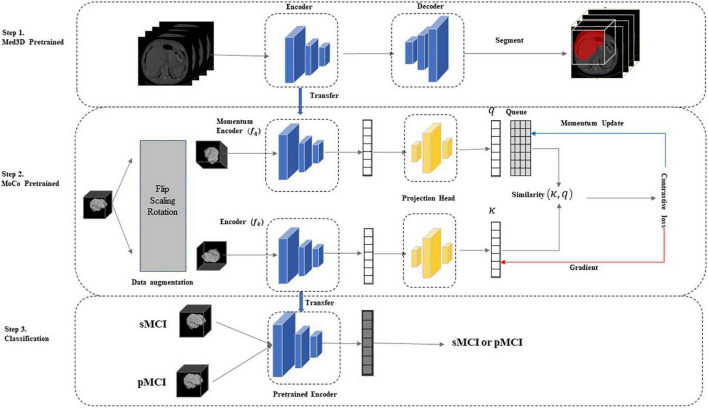
The two-stage model. In step 1, we used the Med3D (Chen et al., 2019) pre-trained model to initialize network weights and learn the common medical image features. In step 2, we performed contrastive learning on unlabeled sMCI and pMCI samples using the improved MoCo to update the network weights, further increasing the correlation between the target and source domains and learning the specific medical features of the sMCI/pMCI classification task. Finally, the network was fine-tuned using labeled sMCI/pMCI samples to achieve sMCI and pMCI classification. MoCo, Momentum Contrast; pMCI, progressive mild cognitive impairment; sMCI, stable mild cognitive, impairment.

As indicated in Figure 1, our two-stage transfer learning model was divided into three main parts. In our framework, we did not directly transfer trained model on natural image sets or other medical image sets to our research such as previous studies, mainly for the following reasons: at first, the adoption of 3D CNN in this study to preserve more spatial information limits direct transfer learning from 2D natural images; second, the different components of medical image sets may harm the performance. For instance, the Med3D is composed of MRI and CT of brains, lungs, chests, and other organs (Chen et al., 2019), while our MCI data set only includes brain MRI data. The details of each step were described next.

### Dataset and Data Preprocessing

Data used in our study were obtained from the Alzheimer’s Disease Neuroimaging Initiative (ADNI) database, which is the largest open-access AD database with wide popularity in AD-related research. It was launched in 2003 by the several public and private organizations to measure the progression of MCI and early AD by medical image (such as MRI, PET), genomics, biological markers, and neuropsychological assessments (Jack et al., 2008). More information can be found at http://adni.loni.usc.edu/.

As defined in this study, participants with MCI at baseline who developed or did not develop AD within 3 years were referred to as pMCI and sMCI, respectively. To prevent data leakage, only participants’ baseline data were selected in this study. Finally, data from 577 MCI subjects (Means ± std age = 73.08 ± 7.25 years) were included in our study, and 297 of the MCI was classified as sMCI (51.5%) and the rest 280 were pMCI (48.5%). The demographics and the mini-mental state examination scores (MMSEs) information of subjects is summarized in Table 1. Differences in the median age and gender between groups were tested using ANOVA and Fisher’s exact test, respectively. These two interactions yielded no statistically significant results (*p* > 0.05).

**TABLE 1:** A summary of the demographic and clinical information of participants.

	Number	Age (years old)	Sex(M/F)	MMSE
sMCI	297	72.2 ± 7.4[55.0,88.4]	174/123	28.0 ± 1.7[23,30]
pMCI	280	73.9 ± 6.9[55.2,88.3]	172/108	26.8 ± 1.8[21,30]

*Values are presented as Means ± Standard Deviation [Range]; M, Male; F, Female; MMSE, Mini-Mental State Examination.*

**FIGURE 2:**
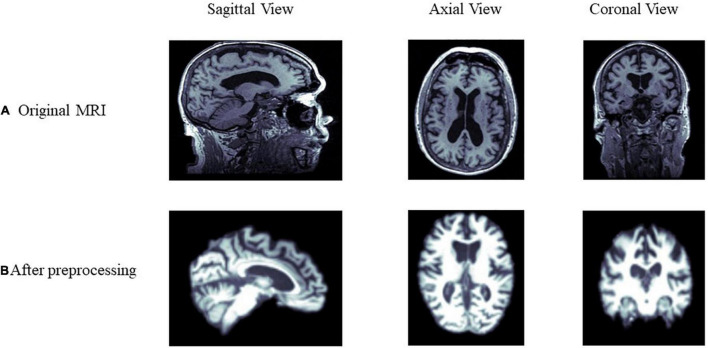
Comparison of MRI data before and after preprocessing. **(A,B)** Show the differences before and after preprocessing from the sagittal, axial, and coronal view of the brain, respectively.

All 1.5T and 3T structural MRI of the participant were downloaded. The detailed information of MRI, such as scanner protocols, can be found at http://adni.loni.usc.edu/methods/documents/mri-protocols/. Data are preprocessed using FSL^1^ with three main steps: bias field correction using the N4 algorithm (Tustison et al., 2010); affine linear alignment of scans onto the MIN152 atlas; skull stripping of each image for 129 × 145 × 129 voxels. Figure 2 shows the difference before and after preprocessing of the MRI from the same sample.

### Network Weight Initialization Using Med3D

Many studies have demonstrated that using transfer learning parameter initialization can significantly improve the performance of models compared to training from scratch (Afzal et al., 2019; Mousavian et al., 2019; Naz et al., 2021). This study selected the Med3D network and its pretrained weights on eight segmented datasets (Chen et al., 2019).

**FIGURE 3:**
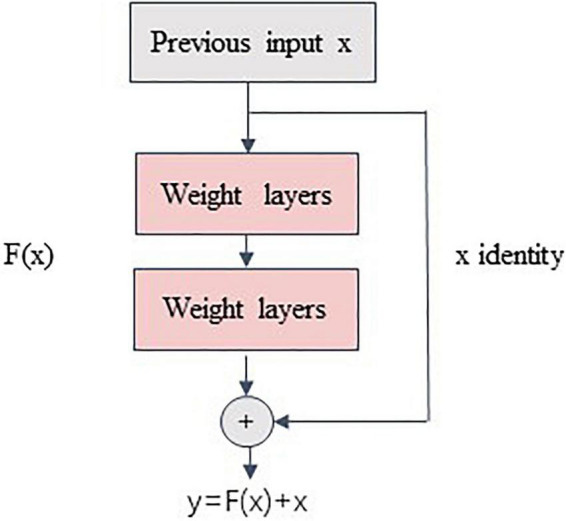
Residual block. The ResNet’s Core Modules.

The authors of Med3D integrated data from eight medical segmentation datasets to create the 3DSeg-8 dataset, which contains multiple modalities (MRI and CT), target organs (brain, heart, pancreas), and pathological conditions (CodaLab, 2021 Competition; Menze et al., 2015; Tobon-Gomez et al., 2015; Medical Segmentation Decathlon, 2021). Med3D uses a standard encode–decode partition structure, where the encoder uses the ResNet family. The main idea of ResNet is to introduce the residual block in the network, as illustrated in Figure 3, where F(x) is the residual mapping and X is the identity mapping, also called “shortcut.” This helps train a deeper network to achieve higher accuracy without vanishing or explosion of the gradient. Notably, Med3D uses a parallel format for model training, which means the same encoder is used for eight datasets, and the decoder is composed of 8 branches accordingly in parallel. This allows the decoder to adapt to different organizational segmentation targets, while the encoder can learn universal features. Figure 1, Step1 depicts the Med3D structure. Med3D pretrained models can be used for classification, detection, and segmentation. We used the parameters of the models pretrained by the 3DSeg-8 dataset for the initialization of our network. Transfer learning strategy effects were evaluated in various ResNet networks in Med3D, including, ResNet-18, ResNet-34, ResNet-50, ResNet-101, ResNet-152 (He et al., 2016).

### Transfer Learning Using Contrastive Learning

We extracted general 3D medical image features by Med3D (Chen et al., 2019). However, while Med3D includes MRI and CT of brains, lungs, chests, and other organs, whereas our MCI data set only include brain MRI data, there are still domain transfer concerns between the dataset of Med3D and MRI of sMCI and pMCI (Chen et al., 2019), while our MCI data set is only composed of brain MRI data. Contrastive learning, a special unsupervised learning method with a supervisory function, was introduced in this study to further increase the correlation between the target and source domains. The labels of contrastive learning are generated by the data itself rather than by manual labeling (Liu et al., 2020). It uses unlabeled data to train models and learn embeddings of the data by maximizing the consistency between different augmented views of the same sample and minimizing the consistency between different samples through a contrast loss function (Tian et al., 2020).

Currently, there are various representative contrastive learning methods, such as MoCo (He et al., 2020), SimCLR (Chen T. et al., 2020), and PIRL (Misra and van der Maaten, 2020). Because of the sample size and computational resources constraints, we chose MoCo as our pretraining model in our study. Unlike the end-to-end gradient update of SimCLR, MoCo introduces a dynamic queueing dictionary, which is updated by adding new training batches to the queue while removing the oldest ones from the queue according to the first-in-first-out principle and keeping the length of the queueing dictionary unchanged. This approach allows MoCo to obtain good training results with small batch size.

Given and preprocessed sample _x_, contrastive learning obtains two augmented samples _x_q__ and _x_k__ by data augmentation of sample *_  x. x_q__* and *_x_k_ _* are referred to as query data and key data, respectively. q and k are the latent representation of _*x_q_*_ and _*x_k_*_ using a query encoder *_q=f_q___(_  _x_q__;_θ_q___)_* and key encoder *_k=f_k___(x_k__;_θ_k___)_* with weight *_θ_q__* and *_θ_k__*, respectively. If the query and the key belong to the same sample, it is marked as a positive pair _*k+*_. Otherwise, it is a negative sample pair _*k–*_. The auxiliary task in contrastive learning is: given a pair (_    x_q__, _      x_k__), determining whether it is a positive or negative sample pair and making the positive samples closer together and the negative samples further apart.

Give a query q. MoCo applies a queue storing a set of keys k from different samples, including one _*k+*_ and several _*k–*_. The contrastive loss can be defined as:


(1) $${ℒ}_{q,k+,\left\{k^{-}\right\}}=-\text{log}\,\frac{\exp\left(q\cdot k^{+}/\tau \right)}{\text{exp}\,\left(q\cdot k^{+}/\tau \right)+\sum_{k^{-}}\text{exp}\,\left(q\cdot k^{-}/\tau \right)}$$

Here, _τ_ is the temperature parameter. The model updates the encoder weights by minimizing the contrastive loss.

In MoCo, the key encoder is neither updated by back-propagation nor copied from the query encoder. Still, a running average of the key encoder is used, which is known as a momentum encoder. The updating of _θ_q__ and _θ_k__ can be formulated as:


(2) $$\begin{array}{c} \theta_{q}\leftarrow \theta_{q}-\alpha \,\frac{\partial ℒ}{\partial \theta_{q}}\\ \theta_{k}\leftarrow m\,\theta_{k}+\left(1-m\right)\,\theta_{q} \end{array}$$

Here, m _∈_ [0, 1) is the momentum coefficient. As in Eq. (2), _θ_k__ is updated more smoothly than _θ_q__ which is updated by back-propagation.

**FIGURE 4:**
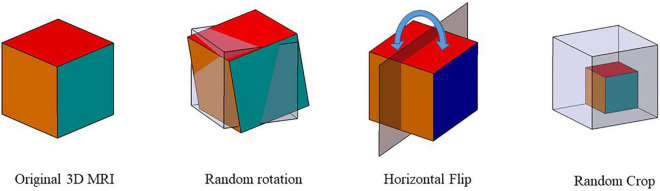
Data augmentation schematic. Three data augmentation were used to augment the data size: random rotation, horizontal flip, and random scaling.

It was shown that data augmentation methods such as Gaussian blur and color distortion for natural images might not be applicable in the medical image. For example, Gaussian blur on MRI can potentially change the label of the data. Therefore, we improved the data augmentation method in MoCo by using random rotation (± 10°), horizontal flip, and random scaling (± 10%) on 3D MRI. In detail, we rotated images at any angle between –10° and +10° along the three axes. Random scaling was also applied to randomly scale the image by ± 10%. If the image size is larger than the size of the original image, the same volume of the original image is extracted by cropping the center region of the image. If the volume is less than its original size, it is filled with 0 in the reduced region. Figure 4 shows the schematic diagram of the three data augmentation methods.

We performed MoCo on the full unlabeled MRI data using the Med3D pretrained ResNet as the encoder. In addition, as it is shown that adding a projection head helps to learn feature representation better (Chen T. et al., 2020; Chen X. et al., 2020), we added a projection head, as shown in Figure 1. We used two MLP with 128-D hidden layers and a ReLU activation function as the projection head as:


(3) $$\begin{array}{c} z_{q}=g\,\left(q\right)=W^{2}\,\sigma \,\left(W^{1}\,q\right)\\ z_{k}=g\,\left(k\right)=W^{2}\,\sigma \,\left(W^{1}\,k\right) \end{array}$$

where *_W^1^_* and *_W^2^_* represent the hidden and output layer weights, and _σ_ is the ReLU activation function.

### Classification

The last step of our model is classification, where the labeled data were divided into training, validation, and test sets to fine-tune the pretrained encoders. We added a linear classifier to the frozen backbone model to complete the classification of sMCI and pMCI as proposed by Chen X. et al. (2020).

### Model Evaluation

We first used different ResNet, including ResNet-10, ResNet-34, ResNet-50, ResNet-101 as our two-stage model backbone network, and selected the ResNet with the best performance (ResNet-50, see in results) as our backbone in the following comparative experiment.

#### Evaluation of Transferring Learning Strategies

We first conducted the following comparative experiments with different transfer learning strategies. All the models in different transfer learning strategies used the best ResNet selected by the first experiment.

λ Med3D, pretrained ResNet using Med3D and fine-tuned the model to complete the classification of sMCI and pMCI.

λ MoCo, random initialization of weights, followed with the modified MoCo in the method to train the ResNet without using sMCI/pMCI labels. Then fine-tune the model using labeled data and do the classification.

λ Only ResNet, random initialization of weights, and training ResNet from scratch.

λ Med3D+MoCo, our two-stage model.

#### Comparison With Previous Studies

To comprehensively understand the performance of our method, we reviewed the state-of-the-art literature, which utilized deep learning to predict the conversion from MCI to Alzheimer’s using MRI. We selected studies that achieved criteria for a fair comparison, including (1) only used MRI. (2) published in the last 3 years. (3) the data were from ADNI.

We selected five evaluation metrics to evaluate our model accurately. (1) Accuracy (Acc) is used to measure the proportion of correctly classified samples. (2) Sensitivity (Sens), also known as the true positive rate, is the proportion of predicted positive results that are true positives. (3) Specificity (Spec) is the proportion of correctly identified negatives. (4) F1-score (F1) is the reconciled average of sensitivity and retrieval. (5) Area Under ROC Curve (AUC) represents how the false-positive rate increases with the true-positive rate and increases the area under the characteristic curve. The aforementioned evaluation metrics were calculated based on True Positive (TP), False Positive (FP), False Negative (FN), and True Negative (TN), and these four indicators form a confusion matrix.

In our study, sMCI and pMCI were referred to as positive and negative examples, respectively. We can calculate accuracy, sensitivity, specificity, and F1 as follows:


(4) $$A\,c\,c=\frac{T\,P+T\,N}{T\,P+T\,N+F\,N+F\,P}$$

(5) $$S\,P\,E=\frac{T\,N}{T\,N+F\,P}$$

(6) $$S\,E\,N=\frac{T\,P}{T\,P+F\,N}$$

(7) $$𝔽\,1=2\times \frac{\mathrm{TP}}{\left(2\times \mathrm{TP}+\mathrm{FP}+\mathrm{FN}\right)}$$

Finally, we used the non-parametric bootstrap to construct each evaluation metrics’ CIs. A total of 10,000 bootstrap replicates were extracted from the test set, and the performance metrics of the model on each bootstrap replicate were calculated. This generated a distribution for each estimate and reported 95% bootstrap percentile intervals (2.5 and 97.5 percentile) (Efron and Tibshirani, 1993).

### Experimental Settings

**FIGURE 5:**
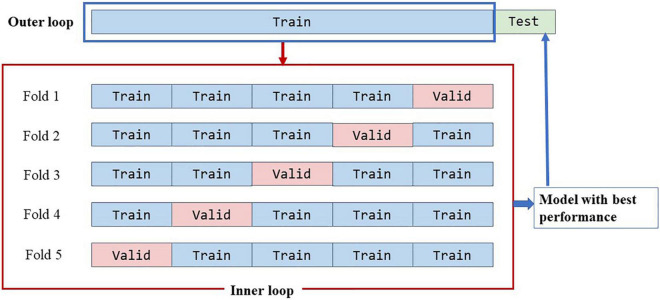
Data spilt strategies.

The model was implemented in PyTorch. We used Stochastic Gradient Descent (SGD) with a weight decay of 0.0001 and momentum of 0.99 as our optimizer. A minibatch size of 16 and a cosine annealing learning rate with an initialized value of 0.01 were used in training. Other hyperparameters are the same as default values in MoCo (He et al., 2020). All unlabeled data were used to train MoCo. We trained the classifier with optimized cross-entropy loss and a learning rate of 0.001 in 100 epochs. The dataset was randomly split into training and testing data with a ratio of 8:2. Optimal hyperparameters were selected using fivefold cross-validation on the training set, and the optimal model was chosen for model evaluation on the test set (Figure 5). All experiments were run on NVIDIA GTX 2080.

## Results

### Results of Different ResNet Models Using a Two-Stage Model

**FIGURE 6:**
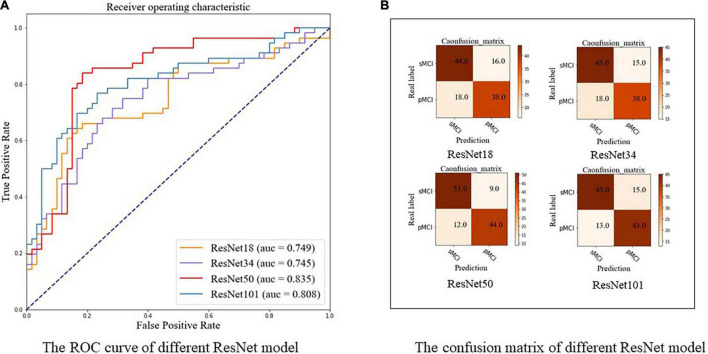
The ROC curve and confusion matrix of different ResNet using our two-stage model. **(A)** The ROC curve. **(B)** The confusion matrix. pMCI, progressive mild cognitive impairment; sMCI, stable mild cognitive impairment.

In this part, we investigated the classification performance of different Med3D pretrained CNN backbones on the test set, including ResNet-18, ResNet-34, ResNet-50, and ResNet-101. As highlighted in Table 2 and shown in Figure 6A, ResNet-50 achieved the best performance with an accuracy of 0.819 and an AUC of 0.835, indicating complex models with more parameters may not always work best. ResNet-50, the model with the best performance, was used in our following experiments. The confusion maps of different ResNet are shown in Figure 6B.

**TABLE 2:** Performance of different ResNet using two-stage model.

Encoder	Acc (95% CI)	Sens (95% CI)	Spec (95% CI)	F1 (95% CI)
ResNet-18	0.707 (0.674, 0.735)	0.679 (0.637, 0.720)	0.733 (0.698, 0.772)	0.691 (0.651, 0.723)
ResNet-34	0.716 (0.690, 0.741)	0.679 (0.644, 0.715)	0.750 (0.716, 0.790)	0.697 (0.670, 0.726)
ResNet-50	**0.819 (0.798, 0.841)**	**0.786 (0.754, 0.821)**	**0.850 (0.815, 0.877)**	**0.807 (0.783, 0.834)**
ResNet-101	0.759 (0.730, 0.783)	0.767 (0.731, 0.808)	0.750 (0.716, 0.785)	0.754 (0.724, 0.779)

*The bold numbers denote the maximum value of each column.*

*Acc, Accuracy; CI, Confidence Interval; F1, F1-score; Sens, Sensitivity; Spec, Specificity.*

### Results of Different Transfer Learning Strategies Using ResNet-50

**FIGURE 7:**
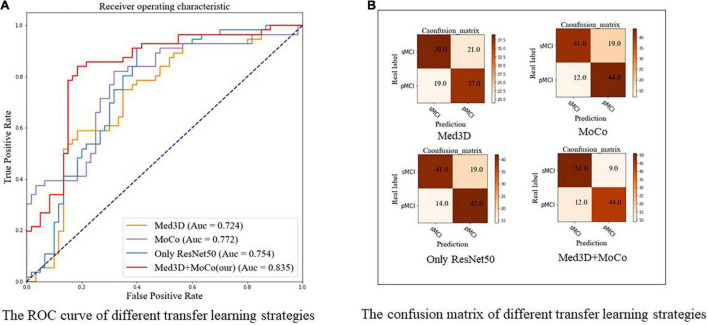
The ROC curve and confusion matrix of different transfer learning strategies using ResNet-50. **(A)** The ROC curve. **(B)** The confusion matrix, respectively. All the models in this figure use ResNet-50. Med3D, pre-trained ResNet-50 using Med3D and fine-tune the model to complete the classification of sMCI and pMCI; MoCo, random initialization of weights, followed with the modified MoCo in the method to train the ResNet50 without using sMCI/pMCI labels. Then fine-tune the model using labeled data and complete the classification; Only ResNet, random initialization of weights and training ResNet from scratch; Med3D+MoCo, our two-stage transfer learning model; MoCo, Momentum Contrast; pMCI, progressive mild cognitive impairment; sMCI, stable mild cognitive impairment.

Table 3 and Figure 7 show the results of the comparison of accuracy, sensitivity, specificity, F1, and ROC for different transfer learning strategies (based on ResNet-50) mentioned in section “Experimental Settings,” where the optimal results are highlighted. As Table 3 and Figure 7A indicate, our method achieves better results compared to other methods in terms of accuracy (0.819), sensitivity (0.786), specificity (0.850), and F1 score (0.807). Similarly, Figure 7A shows ROC curves of different transfer learning strategies, where our method has the best AUC of 0.835 compared with other methods. The confusion maps of different transfer learning strategies are shown in Figure 7B.

**TABLE 3:** Performance of different transfer learning strategies using ResNet-50.

	Acc (95% CI)	Sens (95% CI)	Spec (95% CI)	F1 (95% CI)
Med3D	0.655 (0.630,0.682)	0.661 (0.623,0.702)	0.650 (0.609,0.685)	0.649 (0.619, 0.6768)
MoCo	0.733 (0.704, 0.756)	0.786 (0.747, 0.820)	0.683 (0.651, 0.725)	0.740 (0.712, 0.762)
Only ResNet-50	0.716 (0.694, 0.744)	0.750 (0.712, 0.789)	0.683 (0.656, 0.722)	0.718 (0.694, 0.744)
Med3D+MoCo	**0.819 (0.798, 0.841)**	**0.786 (0.754, 0.821)**	**0.850 (0.815, 0.877)**	**0.807 (0.783, 0.834)**

*The bold numbers denote the maximum value of each column.*

*Acc, Accuracy; CI, Confidence Interval; F1, F1-score; MoCo, Momentum Contrast; Sens, Sensitivity; Spec, Specificity.*

### Results of the Relevant Brain Region

**FIGURE 8:**
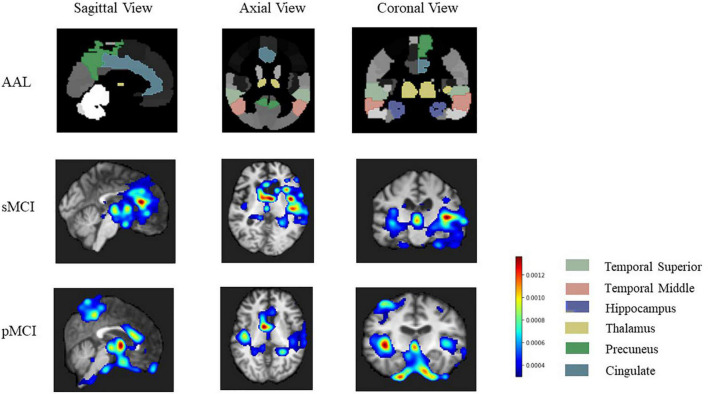
The heatmap of related brain region our model focuses on. The first row is a golden standard of temporal superior, temporal middle, hippocampus, thalamus, precuneus, cingulate in automated anatomical labeling (AAL2, http://www.gin.cnrs.fr/en/tools/aal-aal2/). The second and third rows show brain regions that our model focuses on more on sMCI and pMCI, respectively. pMCI, progressive mild cognitive impairment; sMCI, stable mild cognitive impairment.

In this study, the 3D Grad-CAM method was used to identify brain regions associated with sMCI or pMCI and improve the interpretability of the model. After weight back-propagation of trained models, we obtained average relevance heatmaps of each class in the test dataset. For comparison, we highlighted temporal superior, temporal middle, hippocampus, thalamus, precuneus, cingulate in the automated anatomical labeling (AAL2)^2^ in Figure 8 first row. Figure 8 second and third rows show each class’s last convolutional layer’s attention heatmap. As shown in Figure 8, the hippocampus, temporal superior, temporal middle, thalamus, and cingulate are relevant for both sMCI and pMCI. But precuneus is recognized as a unique feature of pMCI.

### Comparisons With Previous Studies

We further used four evaluation metrics to compare our results to previous state-of-the-art deep learning studies on sMCI/pMCI classification published in the last 3 years, including accuracy, sensitivity, specificity, and AUC. Table 4 summarizes the results in relation to the literature, and the best results are indicated by the bold text. In the classification tasks of sMCI and pMCI using deep learning, our method achieves better or comparable classification results in terms of accuracy, specificity, and AUC.

**TABLE 4:** A summarized comparison of state-of-the-art research on MRI using deep learning in sMCI and pMCI classification.

Research	Conversion time	sMCI/pMCI number	Network	Acc	Sens	Spec	AUC
Liu M. et al. (2018)	36 Months	100/164	Landmark detection and 3D CNN	0.77	0.42	0.82	0.78
Liu J. et al. (2018)	18 Months	160/120	Whole brain hierarchical network	0.72	0.75	0.71	0.72
Lin et al. (2018)	36 Months	139/169	2D CNN	0.79	**0.86**	0.68	0.83
Shmulev and Belyaev (2018)	60 Months	532/327	3DResNet/VoxCNN	0.62	0.75	0.54	0.70
Shi et al. (2018)	18 Months	56/43	2D Deep polynomial network	0.79	0.68	0.87	0.80
Basaia et al. (2019)	36 months	253/510	3D CNN	0.75	0.75	0.75	NA
Li et al. (2019)	36 months	95/126	Self-weighting grading biomarker	0.69	0.82	0.51	0.70
Oh et al. (2019)	36 months	101/106	3D CNN + Transfer learning from CN/AD	0.73	0.77	0.71	0.79
Spasov et al. (2019)	36 months	228/181	3D CNN	0.72	0.63	0.81	0.79
Abrol et al. (2020)	24 Months	409/217	2D Multiscale Deep Neural Network	0.75	0.73	0.76	0.71
Gao et al. (2020)	36 Months	129/168	3D CNN + Transfer learning for AD age prediction	0.81	0.76	0.77	0.76
Pan et al. (2020)	18 Months	173/105	CNN and ensemble learning	0.62	NA	NA	0.59
Wen et al. (2020a)	36 Months	298/295	3D CNN	0.74	0.80	0.68	NA
Bae et al. (2021)	36 Months	222/228	3D ResNet29 + Transfer learning from CN/AD	**0.82**	0.72	NA	0.83
Guan et al. (2021)	36 Months	401/197	3D CNN	0.79	0.55	0.84	0.78
Zhang J. et al. (2021)	18 Months	251/162	3D DenseNet + Attention	0.79	0.75	0.82	**0.86**
Our	36 months	297/280	3D ResNet and transfer learning from self	**0.82**	0.79	**0.85**	0.84

*The bold numbers denote the maximum value of each column.*

*Acc, Accuracy; AD, Alzheimer’s disease; AUC, Area Under Curve; F1, F1-score; NC, Normal control; pMCI, progressive mild cognitive impairment; Sens, Sensitivity; Spec, Specificity; sMCI, stable mild cognitive impairment.*

## Discussion

This study proposed a two-stage method that combined contrastive learning and transfer learning for predicting conversion from MCI to AD based on MRI. Pretrained models from sizeable medical image datasets were used to initialize the model parameters and obtain general imaging features. Training on unlabeled target datasets using contrastive learning was used to get target imaging features. At last, the network was fine-tuned using the labeled target dataset to complete the classification. In addition, 3D Grad-CAM was used to highlight brain regions potentially associated with pMCI/sMCI classification. We demonstrated the validity of our model through multiple evaluation experiments. The experiments showed that our model outperformed both transfer learning and contrastive learning individually and achieved better or comparable results than previous state-of-the-art studies.

Several factors improve the performance of our classification model. The first contribution is the proposal of a two-stage model. Table 3 shows that the classification accuracy of ResNet-50 trained from scratch on sMCI and pMCI is 6.03% higher than that of ResNet-50 pre-trained in Med3D, implying that direct transfer learning for two data sets that are not highly correlated does not always achieve good results, and may even result in a negative transfer. The performance of transfer learning is affected by the various factors such as the size of pretrained samples, the relevance of the source and target domains. Thus, not all the transfer learning can improve the model’s performance (Huh et al., 2016; Zhuang X. et al., 2019; Alzubaidi et al., 2020, 2021; Mustafa et al., 2021). For example, Alzubaidi et al. (2021) found that the model trained from scratch performed better than those pretrained by ImageNet using three different medical imaging datasets. This observation inspired us to develop a two-stage model. Our two-stage model is sample efficient when compared with existing transfer learning-based models for sMCI and pMCI classification (Oh et al., 2019; Gao et al., 2020; Bae et al., 2021). In each of these studies, additional AD and NC samples were collected for training. But our model does not require additional data collection, makes full use of each sample, and produces better or equivalent results. For example, Bae et al. (2021) developed a transfer learning model based on 3D ResNet29. In the source task, the model is pretrained using MRI scans of 2,084 normal samples and 1,406 AD samples. Then they used pMCI and sMCI samples to fine-tune the model to accomplish the target task of classifying pMCI and sMCI. In comparison to our results, they got the same accuracy but lower AUC.

To the best of our knowledge, we are the first to use the MoCo pretrained model for sMCI and pMCI classification. Compared with the models, only pretrained by Med3D and ResNet-50 trained from scratch, our method improved accuracy by 16.4 and 10.3%, respectively, further demonstrating the importance and efficiency of including contrastive learning into our method. Pretraining by contrastive learning allows the model to have a feature representation with better generalization at the same domain of the target task (Sun et al., 2019). Recent studies have shown that fine-tuning on a well-trained contrastive learning model can achieve comparable or even better results than fully supervised learning (Wu et al., 2018; Zhuang C. et al., 2019), which is consistent with our findings. In addition, one of the critical factors limiting the performance of contrastive learning is the slow convergence rate (Chen T. et al., 2020; Chen X. et al., 2020; Tian et al., 2020). As shown in Table 3, compared with MoCo trained from scratch, our method improved accuracy by 6.89%, which indicates that transfer learning can accelerate the convergence of the MoCo model and improve the model performance. MoCo and transfer learning can reinforce and complement one other.

In addition, our model uses complete 3D MRI as model input. Unlike models using 2D slices, the 3D model makes full use of the spatial information of the brain to improve the accuracy of the model (Wen et al., 2020b). Furthermore, some previous studies used feature engineering or cherry-picked regions of interest as input features (Gerardin et al., 2009; Ahmed et al., 2014; Basaia et al., 2019), which ignored the contributions of other features in the model, resulting in information loss in some cases. For example, Basaia et al. (2019) chose brain gray matter to train the model, neglecting the role of cerebrospinal fluid or white matter in early diagnosis of AD (Jack et al., 2010; Weiler et al., 2015). Our model differs from the previous studies by using an end-to-end model to learn from all possible features in medical images, which improves model performance.

In Figure 8, the hippocampus, temporal, and thalamus are highlighted in both sMCI and pMCI. Hippocampus and amygdala in the middle temporal lobe have been considered as crucial brain regions for the diagnosis of early AD (Visser et al., 2002; Braak and van Braak, 2004b; Burton et al., 2009; Costafreda et al., 2011). The hippocampus is essential for memory formation, and the recent studies have found that the hippocampus atrophy of pMCI is more pronounced than sMCI (Devanand et al., 2007; Risacher et al., 2009; Costafreda et al., 2011). Similarly, the amygdala, which is primarily responsible for emotion and expression, is intimately linked to emotional changes of AD, such as anxiety and irritability (Unger et al., 1991; Poulin et al., 2011). Thalamic damage is associated with decreased body movement and coordination, attention, and awareness in AD (Braak and van Braak, 2004a; de Jong et al., 2008; Cho et al., 2014; Aggleton et al., 2016). In addition to the hippocampus and temporal, which have been widely studied in AD, our heatmap also reveals that pMCI is also closely related to the precuneus with high-level memory and cognitive functions, which is in line with the previous studies (Whitwell et al., 2008; Bailly et al., 2015; Perez et al., 2015; Colangeli et al., 2016; Kato et al., 2016; Zhang H. et al., 2021). The results, as mentioned earlier, further indicate that some structural brain region abnormalities play an important role in predicting early AD. In summary, our discovery of important brain regions is supported by abundant literature, which helps construct a more comprehensive brain biomarker atlas to predict MCI progression.

## Conclusion

In conclusion, our two-stage model increases both the accuracy of early AD detection as well as the transparency of the model. Notably, a comprehensive comparison of different 3D ResNet networks provides references for related research. Furthermore, the combination of transfer learning and contrastive learning solves the negative transfer problem and alleviates the model overfitting problem due to a lack of medical data. Notably, it also substantially improves the diagnostic performance of this tricky classification problem in neuroscience. Our model only uses low-invasive, low-cost, and widely available MRI data, which significantly expands the application scenarios of the model.

However, this study also has some limitations that merit additional exploration. First of all, we will explore more options for the model’s various modules, such as different data augmentation methods and pretrained models on model effectiveness. When a larger dataset becomes available, we will also continue to validate our model. At final, it is worth noting that a direct comparison of different methods using the same evaluation metrics is straightforward but may not be the optimal solution. Factors such as sample size, dataset split strategy, sMCI, and pMCI definitions, and test data selection can have an impact on model outcomes. A more statistically robust comparison should be proposed in our future studies. Despite these limitations, our model provides a new solution to avoid overfitting because of the insufficient medical data and allows early identification of AD.

## Author’s Note

The numerical calculations in this article have been done on the supercomputing system in the Supercomputing Center of Wuhan University.

## Data Availability Statement

The original contributions presented in the study are included in the article/supplementary material, further inquiries can be directed to the corresponding author/s.

## Author Contributions

PL and LL designed the study and wrote the article. PL implemented the algorithm and preprocessed the data. LH, NZ, HL, and TT provided critical suggestions. All authors contributed to the article and approved the submitted version.

## Conflict of Interest

The authors declare that the research was conducted in the absence of any commercial or financial relationships that could be construed as a potential conflict of interest.

## Publisher’s Note

All claims expressed in this article are solely those of the authors and do not necessarily represent those of their affiliated organizations, or those of the publisher, the editors and the reviewers. Any product that may be evaluated in this article, or claim that may be made by its manufacturer, is not guaranteed or endorsed by the publisher.

## Footnotes

1
https://fsl.fmrib.ox.ac.uk/

2
http://www.gin.cnrs.fr/en/tools/aal-aal2/
